# Synergistic combination of flavopiridol and carfilzomib targets commonly dysregulated pathways in adrenocortical carcinoma and has biomarkers of response

**DOI:** 10.18632/oncotarget.26050

**Published:** 2018-08-31

**Authors:** Naris Nilubol, Myriem Boufraqech, Lisa Zhang, Kelli Gaskins, Min Shen, Ya-Qin Zhang, Sudheer K. Gara, Christopher P. Austin, Electron Kebebew

**Affiliations:** ^1^ Endocrine Oncology Branch, National Cancer Institute, National Institutes of Health, Bethesda, MD, USA; ^2^ National Center for Advancing Translational Sciences, National Institutes of Health, Bethesda, MD, USA; ^3^ Department of Surgery, The George Washington University School of Medicine and Health Sciences, Washington, DC, USA

**Keywords:** adrenocortical carcinoma, flavopiridol, carfilzomib, X-linked Inhibitor of apoptosis, high-throughput screening

## Abstract

Drug repurposing is an effective approach to identify active drugs with known toxicity profiles for rare cancers such as ACC. The objective of this study was to determine the anticancer activity of combination treatment for ACC from previously identified candidate agents using quantitative high-throughput screening (qHTS). In this study, we evaluated the anticancer activity of flavopiridol and carfilzomib in three ACC cell lines *in vitro* and *in vivo*. Human ACC samples were analyzed for drug-target analysis, and cancer-related pathway arrays were used to identify biomarkers of treatment response. Because flavopiridol is a potent cyclin-dependent kinase (CDK) inhibitor, we found significantly higher CDK1 and CDK2 mRNA expression in three independent cohorts human ACC (p<0.01) and CDK1 protein by immunohistochemistry (p<0.01) in human ACC samples. *In vitro* treatment with flavopiridol and carfilzomib in all three ACC cell lines resulted in a dose-dependent, anti-proliferative effect, and the combination had synergistic activity as well as in three-dimensional tumor spheroids. We observed increased G2M cell-cycle arrest and apoptosis with combination treatment compared to other groups *in vitro*. The combination treatment decreased XIAP protein expression in ACC cell lines. Mice with human ACC xenografts treated with flavopiridol and carfilzomib had significantly lower tumor burden, compared to other groups (p<0.05). We observed increased cleaved-caspase expression and decreased XIAP in tumor xenografts of mice treated with combined agents. Our preclinical data supports the evaluation of combination therapy with flavopiridol and carfilzomib in patients with advanced ACC.

## INTRODUCTION

Adrenocortical cancer (ACC) is a rare and aggressive endocrine malignancy. Most patients present with an incurable disease because of advanced locoregional disease and/or distant metastasis. Although a complete surgical resection may achieve long-term remission, patients with ACC generally have a poor prognosis, with a five-year survival rate of only 16–38% [1, 2]. Despite initial R0 surgical resection, 50–80 % of patients develop recurrent or metastatic disease [3, 4]. There has been no improvement in the survival rate of patients with ACC because there is no effective therapy that provides a durable objective response in patients with advanced and metastatic ACC. The option of systemic therapy in ACC is limited. The current systemic therapy for patients with advanced ACC includes mitotane with or without etoposide, doxorubicin, and cisplatin [5]. However, the response rates are low at only 23% with no overall survival benefit, and the toxic side-effects make it difficult for patients to tolerate [6]. Because of these limitations, there is an urgent need for new treatment options for patients with advanced ACC.

To overcome the prohibitive cost and time needed for developing a new therapy for rare cancerssuch as ACC, we previously demonstrated that drug repurposing using quantitative high-throughput screening (qHTS) of a clinically approved drug library is an effective and efficient way to identify active drugs in rare cancers [7–9]. The combination of active drugs that results in a synergistic effect can further enhance anti-cancer treatment efficacy, and this approach has been used in several hematologic and solid cancers [10, 11]. Because the pharmacokinetic, pharmacodynamic, and toxicity profiles of the drugs are well known, an immediate translation into clinical trials can be performed. In addition, the insight from studying the mechanism of drug action against cancer cells can help improve patient selection and identify markers of treatment response.

In this study, we studied the combination treatment of two active drugs, flavopiridol and carfilzomib (a second-generation proteasome inhibitor), in ACC cells *in vitro* and *in vivo*. A synergistic effect of flavopiridol, a potent cyclin-dependent kinase (CDK) inhibitor, and bortezomib, the first-generation proteasome inhibitor, was demonstrated in a preclinical study of leukemic cells and in a clinical trial of patients with refractory lymphomas[12, 13]. To demonstrate that ACC is a suitable target for flavopiridol, we showed that CDK1 and CDK2 are overexpressed in ACC. Combination treatment resulted in synergistic activity in ACC cell proliferation inhibition and destruction of three-dimensional multicellular aggregates. The combination treatment also increased G2M cell-cycle arrest, induced apoptosis, and decreased X-linked inhibitor of apoptosis (XIAP) *in vitro* compared to vehicle and single drug treatment groups. Mice with human ACC xenografts treated with flavopiridol and carfilzomib had significantly lower tumor burden, and increased cleaved-caspase and reduced XIAP expression in tumor xenografts. Our preclinical data supports the evaluation combination therapy with flavopiridol and carfilzomib in patients with advanced ACC.

## RESULTS

### Flavopiridol and carfilzomib are active in ACC cells

Based on previously published results of qHTS [7], we selected flavopiridol and carfilzomib as they were highly active in ACC cell lines. Flavopiridol was more effective than positive control after 48 hours of treatment (115% efficacy in BD140A cells and 106% efficacy in SW13 cells). Carfilzomib was highly effective with 110% efficacy in BD140A cells and 96% in SW-13 cells after 48 hours of treatment, compared to positive control. Flavopiridol had IC50 of 0.42 μM and 0.13 μM in SW-13 and BD-140A, respectively. Carfilzomib had IC50 of 0.42 μM and 0.02 μM in SW-13 and BD-140A, respectively. These concentrations are well below the maximum serum concentration (Cmax); flavopiridol and carfilzomib in humans were 2.3 μM and 2.9 μM, respectively [14, 15].

### CDK1 and CDK2 are overexpressed in ACC

**Figure 1:**
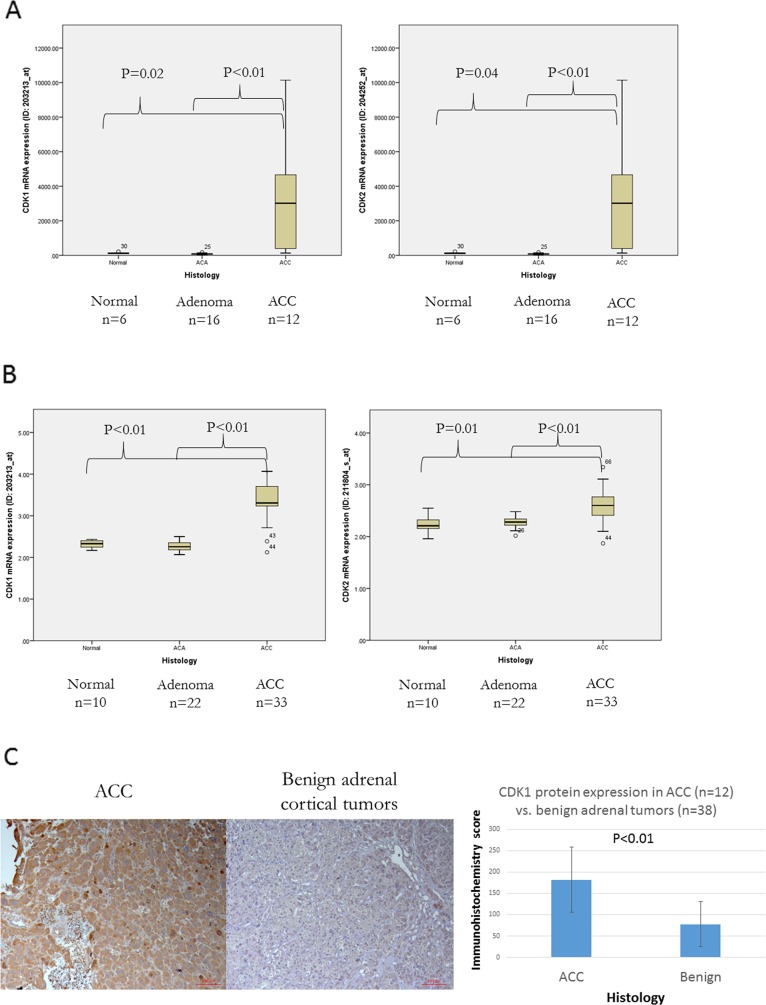
*CDK1* and *CDK2* mRNA Expressions in two independent cohorts using publicly available databases of human ACC samples as compared to normal adrenal tissue and benign cortical adenoma: **(A)** GSE12368 and **(B)** GSE12368. **(C)** CDK1 protein expression in ACC (n=12) vs. benign adrenal cortical tumors (n=38) by immunohistochemistry. Left panel: representative immunohistochemistry image for CDK1 immunostaining. Right panel scoring of immunohistochemistry staining. ACC: adrenocortical carcinoma.

Because flavopiridol is a potent inhibitor of CDK1 and CDK2 [16], we evaluated *CDK1* and *CDK2* mRNA expressions in human ACC samples in publicly available databases. In GSE12368 dataset, *CDK1* and *CDK2* mRNA expressions were significantly higher in ACC (n=12) compared to normal (n=6, p=0.02) and adrenal adenoma (n=16, p<0.01) (Figure 1A). We confirmed this finding in an independent dataset (GSE33371) and found significantly higher *CDK1* and *CDK2* mRNA expressions in ACC (n=33) compared to normal (n=10, p<0.01) and adrenal adenoma (n=22, p<0.01) (Figure 1B). We studied CDK1 protein expression by immunohistochemistry in additional independent samples [ACC (n=12), adrenal cortical adenomas (n=38)], and found that CDK1 expression was significantly higher in ACC (p<0.01) (Figure 1C).

### High CDK1 and CDK2 expressions were associated with aggressive ACC and poor survival in patients with ACC

**Figure 2:**
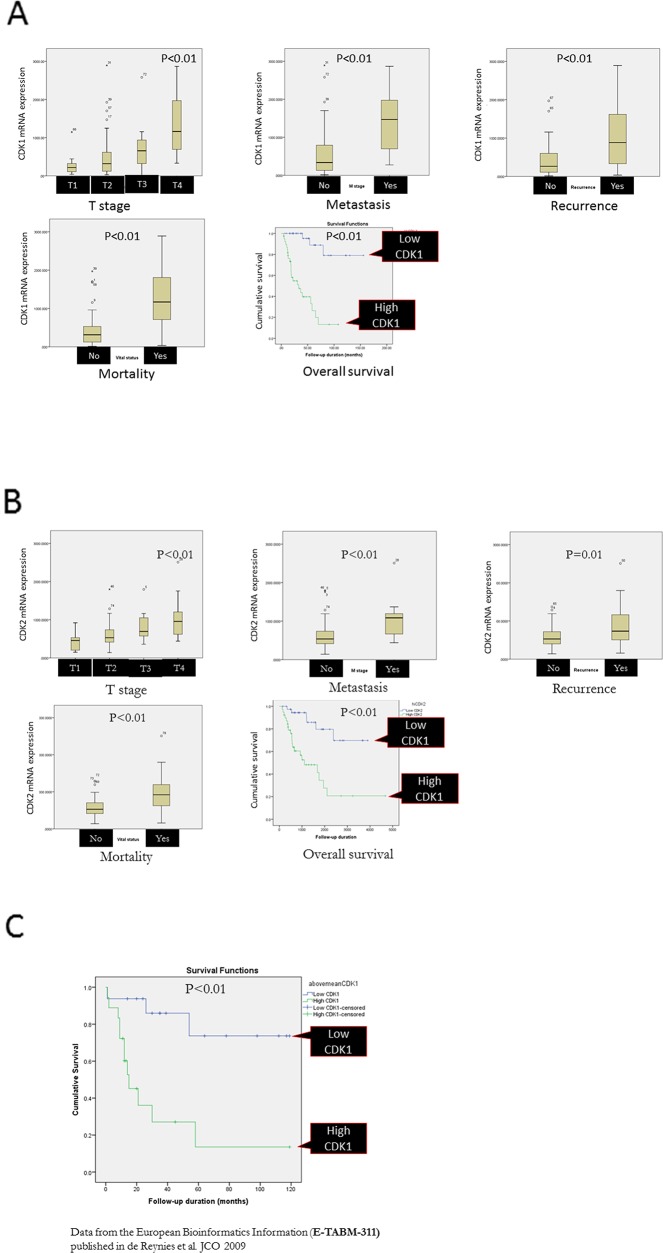
*CDK1* **(A)** and *CDK2* **(B)** mRNA expressions in human ACC samples by clinical features. Data from The Cancer Genome Atlas database (n=92). **(C)** Disease-specific survival in patients with ACC by *CDK1* and *CDK2* mRNA expression [E-TABM-311 database (n=34)].

As CDK1 overexpression is associated with increased cell proliferation, we evaluated the association between *CDK1* and *CDK2* mRNA expressions and adverse clinical features. Using TCGA database, we found higher *CDK1* and *CDK2* mRNA expression in primary T4 stage ACC (p<0.01), distant metastasis (p<0.01), recurrence (p<0.01), and mortality (p<0.01) (Figure 2A and 2B). Patients with high *CDK1* and *CDK2* mRNA expression in primary ACC had significantly shorter overall survival (p<0.01) using the median level as a cutoff (Figure 2A and 2B). To confirm the prognostic significance of *CDK1* and *CDK2* expression in an independent cohort, we analyzed the data from the European Bioinformatics Institute and found shorter disease-specific survival in patients with high *CDK1* and *CDK2* mRNA expression (p<0.01 and p=0.01, respectively) (Figure 2C).

### The combination of flavopiridol and carfilzomib synergistically inhibits cell proliferation, increases G2M cell-cycle arrest, and increases apoptosis in ACC cells

**Figure 3:**
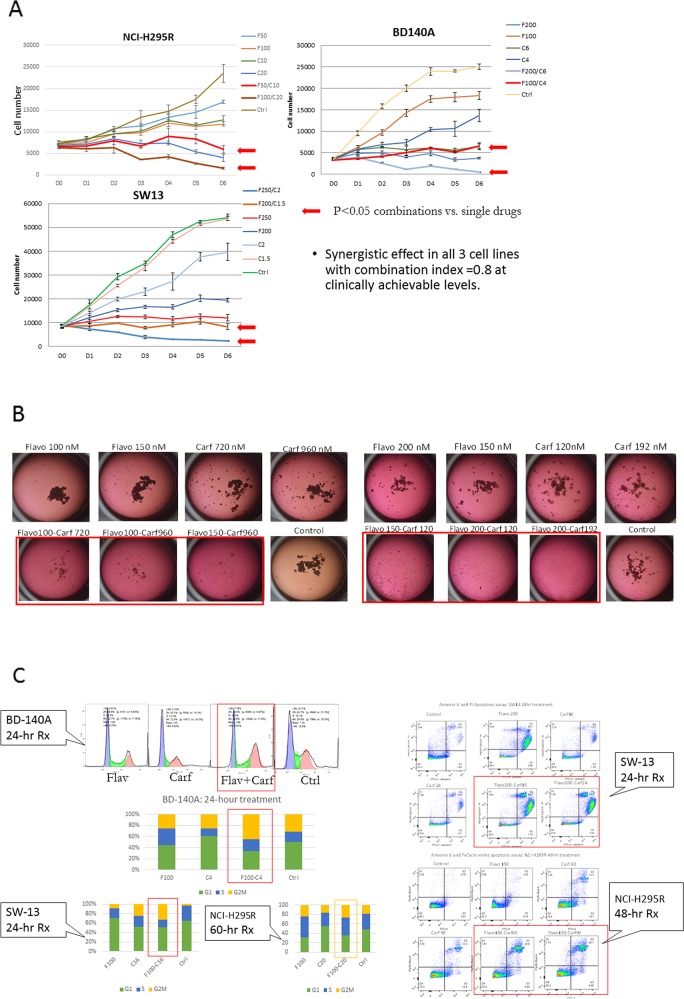
*In vitro* synergistic effects of flavopiridol and carfilzomib in monolayer culture **(A)**, and in three-dimensional multicellular aggregates **(B)** of NCI-H295R and SW13 cells. **(C)** *In vitro* increased G2M cell cycle arrest (left panels) and apoptosis (right panels) in ACC cells treated with the combination of flavopiridol and carfilzomib. Drugs are represented by the initial letters or abbreviated names followed by the concentrations in nM. For example, F100 or Flavo 100 = flavopiridol 100 nM, C10 or Carf 120 = carfilzomib 10 nM or carfilzomib 120 nM, respectively, F50/C10 = the combination of flavopiridol 50 nM and carfilzomib 10 nM, and Ctrl = vehicle control. Duration of treatment is presented in days (D1-D6).

The antiproliferative effect of flavopiridol and carfilzomib was studied in monolayer and three-dimensional cell MCA. First, the combination of flavopiridol and carfilzomib treatment inhibited cell proliferation significantly more than cells treated with single drugs or vehicle control. The anti-proliferative effect of the combination was dose-dependent and a cytotoxic effect resulting in cell death was observed at higher concentrations (Figure 3A). The combination index of several dosage combinations of flavopiridol and carfilzomib in three ACC cell lines were below 1, consistent with synergistic activity. Next, we evaluate the efficacy of the combination treatment in MCAs of NCI-H295R and SW-13 cells. MCAs recapitulate the tumor microenvironment seen *in vivo* that monolayer cell culture cannot provide. After two weeks of treatment, the MCAs of SW-13 treated with flavopiridol and carfilzomib were completely destroyed. MCAs of NCI-H295R treated with the combination of flavopiridol and carfilzomib were present as small and scattered foci at lower dosages and completely destroyed at the higher-dose combinations. In contrast, MCAs of both cell lines treated with single drugs and vehicle control continued to grow and formed aggregates (Figure 3B).

Because we observed an antiproliferative effect and cell death in ACC cells treated with combination flavopiridol and carfilzomib, we studied the effect of the combination on cell-cycle progression and apoptosis. The combination treatment in BD-140A and SW-13 cells at 24 hours increased G2M cell-cycle arrest compared to single drug and vehicle control (G2M phase in BD-140A: flavopiridol, 25.6%; carfilzomib, 25.7%; vehicle, 31.4%; and combination, 44%. G2M phase in SW-13: flavopiridol, 8.9%; carfilzomib, 24.2%; vehicle control, 2.8%; and combination, 32.8%.). G2M phase in NCI-H295R treated with flavopiridol and carfilzomib was higher than the carfilzomib-treated group (27% vs. 16.3%) and vehicle control (27% vs. 18.5%), but only slightly higher than the group treated with flavopiridol alone (24.2% in flavopiridol treated group vs. 27.0% in the combination group).

Because we observed cell death in monolayer and multilayer cell culture models treated with the combination of flavopiridol and carfilzomib, we analyzed the effect of the combination treatment on cell apoptosis by flow cytometry. After 24 hours of treatment, we found an increased population of late apoptotic SW-13 cells in the combination treatment group (69% in 200nM flavopiridol+16nM carfilzomib, and 63.6% in 200nM flavopiridol+24nM carfilzomib) compared to the single drug (50.7% in 200nM flavopiridol only, 10.7% and 15% in 16nM and 24nM carfilzomib only, respectively) and vehicle control (3.8%) groups (Figure 3C). After 48 hours of treatment, we observed an increased population of late apoptotic NCI-H295R cells in the combination treatment group (29.6% in 150nM flavopiridol+ 60nM carfilzomib and 26.1% in 150nM flavopiridol+90nM carfilzomib) compared to the single drug (2.4% in 150nM flavopiridol only, 13.7% and 13.3% in 60nM and 90nM carfilzomib only, respectively) and vehicle control (0.8%) groups (Figure 3C).

### The combination of flavopiridol and carfilzomib reduced XIAP expression

**Figure 4:**
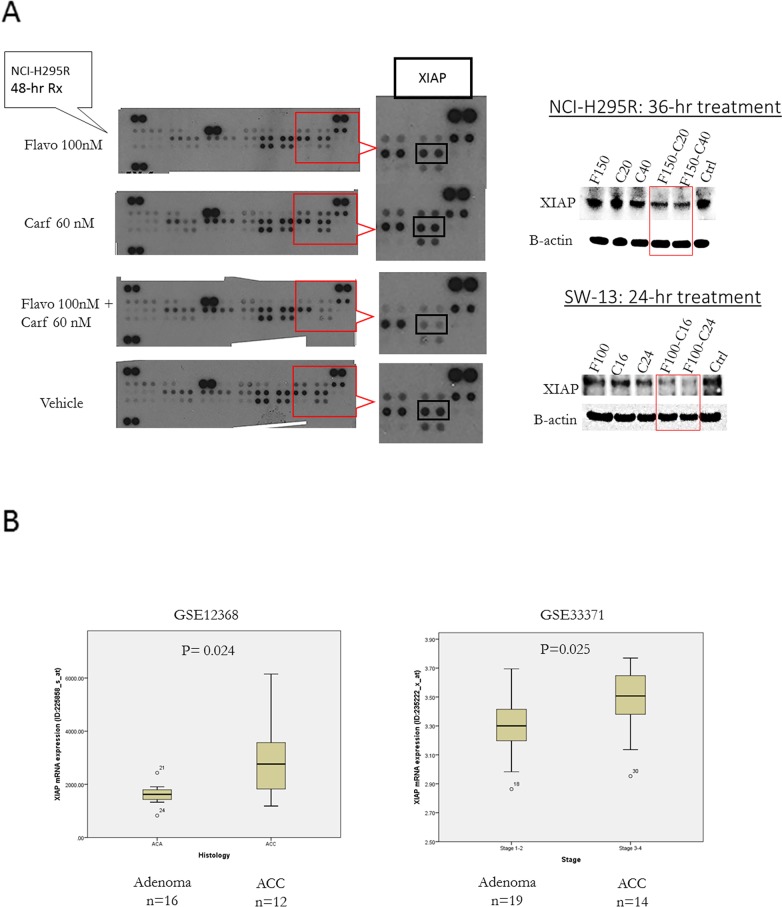
**(A)** XIAP expression in ACC cells treated with flavopiridol and carfilzomib: Human Apoptosis Antibody Array (left panels) and western blots (right panels) with corresponding band densitometry. **(B)** *XIAP* mRNA expression in ACC vs. adrenal cortical adenoma in two independent, publicly available databases of human ACC samples.

To study the mechanism of apoptosis induced by the combination treatment, we used a human apoptotic antibody array that can simultaneously detect the relative expression of 35 apoptosis-related proteins. The combination of flavopiridol and carfilzomib reduced XIAP, anti-apoptotic protein expression compared to single drug and vehicle control groups (Figure 4A). We validated the reduced XIAP expression in NCI-H295R and SW-13 cells treated with the combination of flavopiridol and carfilzomib compared to other groups, by Western blot (Figure 4A). Next, we evaluated the XIAP expression in human tissue samples. XIAP mRNA was overexpressed in ACC compared to cortical adenoma in two independent cohorts (GSE12368: 12 ACC vs. 16 cortical adenoma, p=0.02, and GSE33371: 14 ACC vs. 19 cortical adenoma, p=0.02).

### The combination of flavopiridol and carfilzomib inhibited tumor growth *in vivo* increased apoptosis and reduced XIAP in ACC xenografts

**Figure 5:**
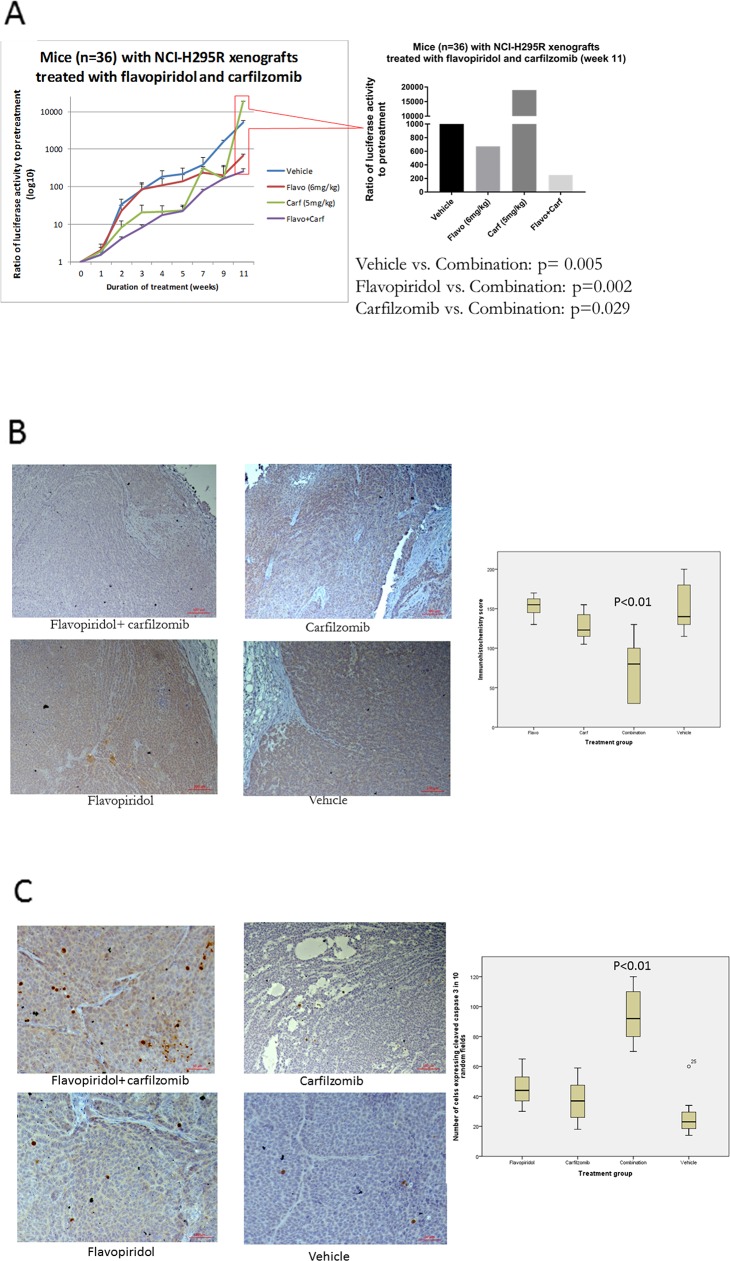
**(A)** *In vivo* efficacy of flavopiridol and carfilzomib in mice (n=36) with NCI-H295R-Luc xenografts. XIAP **(B)** and cleaved-caspase 3 **(C)** expressions by immunohistochemistry in NCI-H295R xenografts treated with flavopiridol, carfilzomib, and vehicle control.

To test *in vivo* efficacy of flavopiridol and carfilzomib, mice (n=36) with subcutaneous NCI-H295R with luciferase reporter were divided into four treatment groups. After 11 weeks of treatment, the luciferase activity from ACC xenografts in mice treated with the combination of flavopiridol and carfilzomib were significantly lower than other groups (Figure 5A, graph is presented in log10 scale). Because the growth of subcutaneous human ACC xenografts in several mice treated with vehicle control and with carfilzomib reached the endpoint for euthanization for a humane reason before the mice died, we euthanized all animals at the same time to accurately assess the effect of treatments. Thus, the effect on survival cannot be assessed in this xenograft model. Next, we studied the effect of the treatments in ACC xenografts. Because we observed reduced XIAP expression in ACC cells treated with flavopiridol and carfilzomib, we assessed XIAP expression in ACC xenografts by immunohistochemistry and found lower expression of XIAP in ACC xenografts treated with the combination of flavopiridol and carfilzomib compared to other groups (p<0.01) (Figure 5B). We found significant increased cleaved caspase-3 in ACC xenografts of mice treated with the combination of flavopiridol and carfilzomib, compared to other groups (p<0.01) (Figure 5C). Mice tolerated the treatment with no significant toxicity.

We analyzed the efficacy of the combination treatment by the initial tumor burden measured by a luciferase activity. Using the median luciferase activity prior to the treatment as a cutoff, we observed a significantly lower tumor growth rate (the ratio of luciferase activity in week 11 to week 0) in the group with higher initial tumor burden (n=6) compared to the group with lower initial tumor burden (n=9) (2.19 vs. 153.7, p=0.018).

## DISCUSSION

In this study, we studied combination treatment with flavopiridol and carfilzomib in ACC cells, *in vitro* and *in vivo*. Because flavopiridol is a potent CDK inhibitor, we studied the expression of CDK1 and CDK2 in human ACC samples. We found that ACC overexpressed CDK1 and CDK2 in multiple independent cohorts. In addition, *CDK1* and *CDK2* mRNA overexpressions were associated with advanced ACC T-stage, recurrence, metastasis, and lower overall survival in two independent cohorts. These findings suggest that ACC is a suitable target for a CDK inhibitor. Next, we validated the qHTS results and found flavopiridol and carfilzomib to have a dose-dependent *in vitro* antiproliferative effect. We observed a synergistic antiproliferative effect and an increase in cell death when ACC cells were treated with both drugs *in vitro*. The combination of flavopiridol and carfilzomib disintegrated ACC MCA. The combination treatment resulted in G2M cell-cycle arrest, increasing apoptosis by effectively reducing XIAP, an anti-apoptotic protein. We found that ACC overexpressed *XIAP* mRNA in two independent cohorts, suggesting that ACC is a suitable target for this combination treatment and XIAP can be used as a marker of response. The results from the *in vivo* treatment of mice with human ACC xenografts confirmed the efficacy of flavopiridol and carfilzomib. This combination effectively reduced XIAP and increased apoptosis in ACC xenografts. These findings support the clinical trial of flavopiridol and carfilzomib in patients with advanced ACC.

Flavopiridol is the first cyclin-dependent kinase inhibitor used in humans. Dysregulation of cyclins and CDK are common in several types of cancer, such as squamous cell carcinoma of the head and neck, esophagus, and uterine cervix; non-small cell lung cancer, breast cancer, soft-tissue sarcomas, and hematologic malignancy [17]. The dysregulation leads to an unchecked proliferation of cancer cells. Flavopiridol is a potent inhibitor of CDK1, 2, 4, and 7 by competitive binding at the ATP-binding pocket [16]. Flavopiridol treatment in human tumor cell lines resulted in drug-mediated cell-cycle arrest and apoptosis [18], but even more remarkably, flavopiridol also induced apoptosis in non-cycling (i.e., G 0–1 phase) cancer cells [19]. Our results were consistent with this observation, as flavopiridol with or without carfilzomib caused G2M cell-cycle arrest in two rapidly dividing ACC cell lines (SW-13 and BD140A), but the effect on cell cycle on the ACC cell line (NCI-H295R) with a slow proliferative rate was minimal. However, we observed increased apoptosis and cell death in NCI-H295R treated with flavopiridol *in vitro* and the effect further increased with the addition of carfilzomib.

Carfilzomib is a second-generation proteasome inhibitor that specifically and irreversibly inhibits the chymotrypsin-like subunit of the 20S proteasome [20]. Carfilzomib is FDA-approved for refractory multiple myeloma. We previously demonstrated the efficacy of bortezomib, the first-generation proteasome inhibitor, in ACC [8]. In the current study, carfilzomib was effective *in vitro* in ACC cells as a single agent. The rationale to combine flavopiridol and carfilzomib derived from a study in human leukemia cells. Dai et al. showed that the NF-kB axis plays a key role in protecting leukemic cells from the lethal consequences of cell-cycle dysregulation by flavopiridol, and that the combination of flavopiridol and MG-132 (a proteasome inhibitor) was synergistically effective in human leukemic cells [21]. The combination treatment resulted in cytochrome C and SMAC/DIABLO release consistent with mitochondrial injuries and induced apoptosis through JNK activation [21]. Our study is the first to demonstrate the synergy of these two drugs in solid malignancy. We did not find cytochrome C and SMAC/DIABLO release in ACC cells treated with flavopiridol and carfilzomib. The mechanism of synergy is the reduction of XIAP and, thus, increased apoptosis. Because CDK1, CDK2, and XIAP are overexpressed in ACC, the combination of flavopiridol and carfilzomib is an appropriate treatment strategy that targets commonly dysregulated proteins in ACC. In addition, we found that patients with ACC overexpressing *CDK1* and*CDK2* had more aggressive tumors with shorter survival. This suggests that such tumors with higher CDK1 and CDK2 may have more responsive tumors to combination treatment. Furthermore, the overexpression of CDKs and XIAP can be used to select patients with advanced ACC to receive this combination treatment and XIAP can also be used as a marker of treatment response. The combination of flavopiridol and bortezomib, another proteasome inhibitor, was found to be a tolerable regimen in patients with heavily pre-treated refractory/recurrent B-cell malignancies in a Phase I clinical trial and resulted in a 33% response rate with 8% complete remission [22]. The other combination therapy that synergistically targets XIAP was demonstrated in non-small cell lung cancer preclinical studies using XIAP-targeting short hairpin RNA (shRNA) and celecoxib [23]. However, the clinical application of shRNA in cancer treatment is limited with no known toxicity or proven efficacy. Our approach using qHTS in the library containing drugs with known toxicity profiles has the advantage in translating into a clinical trial. It is possible that the combination of CDK inhibitor and proteasome inhibitor is more effective in tumors with higher cell proliferation rate because we observed a significantly lower growth rate in mice with higher initial tumor burden receiving the combination treatment. Further studies to assess the mechanisms of resistance in tumors with higher growth rate will be performed.

In summary, flavopiridol and carfilzomib were identified by qHTS as effective novel drugs in ACC. The combination resulted in a synergistic anti-proliferative effect and increased apoptosis via G2M cell-cycle arrest and reduced XIAP levels, *in vitro* and *in vivo*. We found that ACC overexpressed CDKs and XIAP, and that *CDK1* and *CDK2* overexpression can be used as adverse prognostic markers. Our data supports the evaluation of combination treatment with flavopiridol and carfilzomib in a clinical trial in patients with advanced ACC.

## MATERIALS AND METHODS

### Gene expression profiling of ACC

We analyzed publicly available, genome-wide expression data (GSE12368, GSE33371, Gene Expression Omnibus, The National Cancer for Biotechnology Information [24]) to study the messenger RNA expression of *CDK1* and *CDK2* in human ACC samples compared to adrenal adenoma and normal adrenal tissue. To study the association between *CDK1* and*CDK2* messenger RNA expression and clinical characteristics in patients with ACC, we analyzed data from The Cancer Genome Atlas (ACC cohort, n=92) [25] and from the European Bioinformatics Institute (E-TABM-311, n-34) [26].

### ACC cell culture

NCI-H295R and SW-13 cells were grown and maintained in DMEM supplemented with 2.5% Nu-Serum (BD Biosciences, San Jose, CA), and 0.1% ITS premix (BD Biosciences, San Jose, CA). Both cell lines were purchased from American Type Culture Collection™ (Manassas, VA). BD140A cells, kindly provided by Drs. Kimberly Bussey and Michael Demeure (TGen, Phoenix, AZ), were cultured in RPMI supplemented with 1% L-glutamine (Gibco), 1% penicillin-streptomycin (Gibco), and 10% FBS (Invitrogen). The cell lines were authenticated using short tandem repeat profiling. Cells were routinely subcultured every three to four days and maintained in a 5% CO2 atmosphere at 37 C. NCI-H295R cells used to generate human ACC xenograft were transfected with a linearized pGL4.51[*luc2*/CMV/Neo] vector (Promega, Madison, WI) encoding the luciferase reporter gene *luc2* (*Photinus pyralis*) as previously described [27].

### Cellular proliferation assay

NCI-H295R (6×10^3^), SW-13 (4×10^3^), and BD140A (4×10^3^) cells were plated into 96-well clear bottom, black plate (Costar^®^, Corning, NY). We described the culture conditions, drug administration, and technique of cellular proliferation assay in Supplementary Materials.

To examine whether the combination of flavopiridol and carfilzomib was synergistic, we used the automated computerized algorithm (Chou–Talalay method) to calculate the combination index (CI), in which CI=1 indicates an additive effect; CI<1, a synergistic effect; and CI>1, an antagonistic effect [28].

### Three-dimensional multicellular aggregates (MCAs)

The anticancer activity of the candidate drugs were tested in MCAs that mimic solid tumors *in vitro*, in ACC cell lines (NCI-H295R and SW-13 cells) that form MCA. Although monolayer cell cultures can provide a cell-specific response to drugs, this model, however, lacks the tumor microenvironment of three-dimensional solid tumors observed *in vivo*, such as hypoxic tissue areas, regions of differential growth and cell cycling, as well as poor availability of delivered drugs in deeper tumor tissue layers, which can be found in MCAs [17]. The method of MCA culture, treatment, and analysis were described in Supplementary Materials.

### Cell-cycle analysis

Cells plated in 6-well plates were treated with flavopiridol, carfilzomib, the combination, or vehicle for 24–60 hours, depending on the cell line. Cells were trypsinized and fixed for 30 minutes in 70% ethanol at 4°C and stained with 50 mg/mL of propidium iodide containing 100 mg/mL of ribonuclease A. Flow cytometry was performed on a BD FACSCanto I flow cytometer (BD Biosciences, San Jose, CA) using CellQuest software (BD Biosciences, San Jose, CA). Data were generated for at least 20,000 events per sample and analyzed using FlowJo^®^ version 10.2 software (FlowJo, LLC, Ashland, OR).

### Apoptosis assay

SW-13 and NCI-H295R cells were treated in 6-well plates with flavopiridol, carfilzomib, the combination, or vehicle for 24 and 48 hours, respectively. Cells were washed, resuspended in Annexin V binding buffer, and stained with FITC Annexin V per the manufacturer's protocol (#640914, BioLegend, San Diego, CA). Propidium iodine and FxCycle Violet (#F10347, Thermo Fisher Scientific, Waltham, MA) were used to stain late apoptotic and necrotic cells. Flow cytometry was performed on a BD FACSCanto I flow cytometer (BD Biosciences, San Jose, CA) using CellQuest software (BD Biosciences, San Jose, CA). Data were generated for at least 20,000 events per sample and analyzed using FlowJo^®^ version 10.2 software (FlowJo, LLC, Ashland, OR).

### Human apoptosis antibody array

To assess the mechanism of apoptosis, we used a Human Apoptosis Antibody Array, which can simultaneously detect the relative expression of 35 apoptosis-related proteins (Catalog # ARY009, R&D Systems, Minneapolis, MN). NCI-H295R cells treated with flavopiridol, carfilzomib, the combination of flavopiridol and carfilzomib, and vehicle control were lysed and the array membranes were processed according to the manufacturer's protocol.

### Western blot

Cells were lysed in a buffer of 10% SDS and protease/phosphatase inhibitor cocktail (Sigma-Aldrich). The lysates were quantified for protein concentrations using a BCA Protein Assay Kit (Pierce, Life Technologies) per the manufacturer's protocol. The methods of Western Blot was described in Supplementary Materials.

### Immunohistochemistry

Formalin-fixed tissues were embedded in paraffin and cut into 5 μm–thick sections for hematoxylin and eosin (H&E) staining and immunostaining. To assess CDK1 protein expression in human samples, we used an adrenal microarray (AG801, US Biomax, Inc., Rockville, MD) and primary ACC samples (adrenal adenoma, n=38; ACC, n=12). We described the immunohistochemistry technique in Supplementary Materials.

### *In vivo* study in mice with human ACC xenografts

The *in vivo* studies in mice were approved by the National Cancer Institute, National Institutes of Health (NIH), Animal Care and Use Committee. Mice were maintained according to NIH Animal Research Advisory Committee guidelines. A total of 5×10^6^ NCI-H295R cells with luciferase reporter were injected into each flank of a Nuþ/Nuþ mouse (two xenografts per mouse). Tumors were allowed to grow and mice of both sexes were randomized into four treatment groups (nine mice per treatment group). Mice were treated with flavopiridol (6 mg/kg), carfilzomib (5 mg/kg), the combination of flavopiridol (6 mg/kg) and carfilzomib (5 mg/kg), and vehicle control. We used bioluminescence imaging to confirm a deposition of injected cells using the Xenogen *in vivo* imaging system. We described the imaging technique, anesthesia, euthanization, and tumor processing in Supplementary Materials.

### Statistical analysis

We analyzed the gene expression profiling data (GSE12368 and GSE33371) using embedded interactive statistical software (GEO2R). The *P* values were adjusted for false discovery rate using the Benjamini-Hochberg method [29]. The data was present in box-plot, with median in the box that represented the 25^th^ and 75^th^ percentile of data. Error bars demonstrated minimum and maximum values within 95% of data. We used analysis of variance (ANOVA) with post-hoc tests to compare the mRNA expression and *in vivo* luciferase activity between groups. The Kaplan-Meier estimator [30] with the Log-rank (Mantel-Cox) [31] test was used to compare survival between groups. If interactions were found, pairwise comparisons between group levels were calculated with the Bonferroni correction for multiple testing. The Student's t*-*test was used to compare the mean between groups that normally distributed [32]. The Mann–Whitney *U* test was used to compare continuous variables that were not normally distributed [33]. A two-tailed *P* value less than 0.05 was considered statistically significant. Statistical analyses were performed using SPSS version 21.0 for Windows (SPSS, Inc., Chicago, IL).

## SUPPLEMENTARY MATERIALS


