# Supplementary material for: Synergistic combination of flavopiridol and carfilzomib targets commonly dysregulated pathways in adrenocortical carcinoma and has biomarkers of response
Source: Oncotarget. 2018 Aug 31;9(68):33030–42. doi: 10.18632/oncotarget.26050 (PMC6152480; doi:10.18632/oncotarget.26050)
Supplement: Supplementary file 1 [file oncotarget-09-33030-s001.pdf]

Synergistic combination of flavopiridol and carfilzomib targets commonly dysregulated pathways in adrenocortical carcinoma and has biomarkers of response

SUPPLEMENTARY MATERIALS

ACC cell culture

NCI-H295R and SW-13 cells were grown and maintained in DMEM supplemented with 2.5% Nu-Serum (BD Biosciences, San Jose, CA), and 0.1% ITS premix (BD Biosciences, San Jose, CA). Both cell lines were purchased from American Type Culture Collection™ (Manassas, VA). BD140A cells, kindly provided by Drs. Kimberly Bussey and Michael Demeure (TGen, Phoenix, AZ), were cultured in RPMI supplemented with 1% L-glutamine (Gibco), 1% penicillin-streptomycin (Gibco), and 10% FBS (Invitrogen). The cell lines were authenticated using short tandem repeat profiling. Cells were routinely subcultured every three to four days and maintained in a 5% CO2 atmosphere at 37 C. NCI-H295R cells used to generate human ACC xenograft were transfected with a linearized pGL4.51[*luc2*/CMV/Neo] vector (Promega, Madison, WI) encoding the luciferase reporter gene *luc2* (*Photinus pyralis*) as previously described (17).

Cell proliferation assay

NCI-H295R (6×10^3), SW-13 (4×10^3), and BD140A (4×10^3) cells were plated into 96-well clear bottom, black plate (Costar®, Corning, NY). Each well contained 100 µl of culture media with serum. After 24 hours (Day 0), 100 µl of fresh culture media containing doubleconcentrations of the indicated drugs or corresponding vehicles was added into each well.

An additional 100 µl of fresh media containing the 1× concentration of the tested drugs or vehicles was added 48 hours (Day 2) after initial administration to support optimal cell growth. Based on IC50 determined by qHTS, we selected at least five concentrations of flavopiridol and carfilzomib to test the efficacy of various combinations. Each concentration was performed in quadruplicates. CyQuant Cell Proliferation Assay (Invitrogen Corp., Carlsbad, CA) was performed according to the manufacturer's protocol. The cell densities in the 96-well black plates were determined using a SpectraMax M5e 96-well fluorescence microplate reader (Molecular Devices, Sunnyvale, CA) at 485 nm/538 nm. For each drug tested, the experiments were repeated at least three times.

Three-dimensional multicellular aggregates (MCAs)

MCAs of NCI-H295R cells form compact tumor spheroids in suspension culture conditions. We generated NCI-H295R and SW-13 MCAs by plating 1.0×10^5 cells/well into Ultra Low Cluster, 24-well plate (Costar®, Corning, NY) and incubated the cells at 37°C in 5% CO2 for two weeks. The media was changed twice a week. At the beginning of the third week of culture, MCAs were treated with selected combinations and their corresponding single drug and vehicle controls using concentrations based on IC50 from the monolayer proliferation assay below maximum tolerable serum levels. All experiments were performed in triplicates for each testing concentration. SW-13 and H295R MCAs were continuously treated for two weeks. Media containing respective drugs and vehicle control were changed twice a week. MCAs were photographed with a Nikon D5100 (Nikon, Inc., Melville, NY) under a 12.5× magnification microscope (Olympus SZX9 microscope with DF PLAPO 1X-2 lens, Olympus America, Inc., Center Valley, PA).

Western blot

Cell lysates were analyzed in SDS-PAGE and transferred onto polyvinylidene difluoride (PVDF) membranes (Invitrogen, Thermo Fisher Scientific). The membranes were blocked with 5% BSA in TBS-Tween buffer and then incubated with primary antibodies overnight at 4°C. Primary antibodies were anti-XIAP (D2Z8W) rabbit monoclonal antibody at 1:1000 dilution (#14334, Cell Signaling, Inc., Danvers, MA) and B-actin rabbit monoclonal antibody at 1:1000 dilution (#4970, Cell Signaling, Inc., Danvers, MA). The membranes were then incubated with the HRP-conjugated secondary antibodies. Proteins bands were analyzed using enhanced chemiluminescence (ECL) reagent (Pierce, Thermo Fisher Scientific).

Immunohistochemistry

Sections were deparaffinized and rehydrated in serially graded alcohol. Sections were then placed in citrate buffer, and epitope retrieval was performed at 120°C using pressurized steam for 10 minutes. The

endogenous peroxidase activity was blocked with 6% hydrogen peroxide. The primary antibodies were anti-cleaved caspase 3 (Asp175) polyclonal rabbit antibody at 1:300 dilution (#9661, Cell Signaling, Inc., Danvers, MA) and anti-XIAP (D2Z8W) rabbit monoclonal antibody at 1:50 dilution (#14334, Cell Signaling, Inc., Danvers, MA). The sections were incubated at 4°C overnight, then incubated with a biotinylated secondary antibody at 1:150 dilution (Vector Laboratories) for one hour at room temperature. The sections were developed using 3,3'-diaminobenzidine as the chromogen (Elite ABC Kit, Vector Laboratories) and hematoxylin as the counterstain. The sections were dehydrated and mounted with VectaMount Mounting Medium (Vector Laboratories). The slides were viewed under a Zeiss Axio Observer D1 microscope (Zeiss USA, North Chesterfield, VA) and photographs were taken at 200x magnification. The Quickscore (Q) method was used to quantitate the protein expression in immunohistochemistry [1].

In vivo study in mice with human ACC xenografts

A total of 5×10^6 NCI-H295R cells with luciferase reporter were injected into each flank of a Nuþ/Nuþ mouse (two xenografts per mouse). Tumors were allowed to grow and mice of both sexes were randomized into four treatment groups (nine mice per treatment group). Mice were treated with flavopiridol (6 mg/kg), carfilzomib (5 mg/kg), the combination of flavopiridol (6 mg/kg) and carfilzomib (5 mg/kg), and vehicle control. We used bioluminescence imaging to confirm a deposition of injected cells using the Xenogen *in vivo* imaging system. Mice were injected intraperitoneally with 3–4.5 mg of luciferin/mouse 10 minutes prior to imaging. Then the animals were anesthetized in a plastic chamber filled with a 2.5% isoflurane/oxygen/air mixture, and isoflurane anesthesia was maintained using a nose-cone delivery system during imaging. The pseudocolor image representing the spatial distribution of detected photon counts emerging from the active luciferase within each animal was collected. Signal intensity was quantified as the sum of all detected photon counts within a region of interest using IVIS Living Image software (Caliper Life Sciences Inc.). All mice were euthanized by CO_2 inhalation. Tumor xenografts were harvested, flash-frozen in liquid nitrogen, and formalin-fixed. Formalin-fixed tissues were processed into paraffin blocks for H&E and immunohistochemistry staining.

Mice were injected intraperitoneally with 3–4.5 mg of luciferin/mouse 10 minutes prior to imaging. Then the animals were anesthetized in a plastic chamber filled with a 2.5% isoflurane/oxygen/air mixture, and isoflurane anesthesia was maintained using a nose-cone delivery system during imaging. The pseudocolor image representing the spatial distribution of detected photon counts emerging from the active luciferase within each animal was collected. Signal intensity was quantified as the sum of all detected photon counts within a region of interest using IVIS Living Image software (Caliper Life Sciences Inc.). All mice were euthanized by CO_2 inhalation. Tumor xenografts were harvested, flash-frozen in liquid nitrogen, and formalin-fixed. Formalin-fixed tissues were processed into paraffin blocks for H&E and immunohistochemistry staining.

REFERENCE

1. Charafe-Jauffret E, Tarpin C, Bardou VJ, Bertucci F, Ginestier C, Braud AC, Puig B, Geneix J, Hassoun J, Birnbaum D, Jacquemier J, Viens P. Immunophenotypic analysis of inflammatory breast cancers: identification of an 'inflammatory signature'. J Pathol. 2004; 202:265–73.
